# Comparing country risk and response to COVID-19 in the first 6 months across 25 Organisation for Economic Co-operation and Development countries using qualitative comparative analysis

**DOI:** 10.1017/ics.2021.6

**Published:** 2021-03-30

**Authors:** Ian Greener

**Affiliations:** School of Social Work and Social Policy, University of Strathclyde, Glasgow, United Kingdom

**Keywords:** COVID-19, QCA, comparative, testing, typology

## Abstract

This paper explores the contextual and government response factors to the first-wave of the COVID-19 pandemic for 25 the Organisation for Economic Co-operation and Development nations using fuzzy-set qualitative comparative analysis. It considers configurations of: obesity rates; proportions of elderly people; inequality rates; country travel openness and COVID-19 testing regimes, against outcomes of COVID-19 mortality and case rates. It finds COVID-19 testing per case to be at the root of sufficient solutions for successful country responses, combined, in the most robust solutions, with either high proportions of elderly people or low international travel levels at the start of pandemic. The paper then locates its sample countries in relation to existing welfare typologies across two dimensions based on total social expenditure and proportional differences between the GINI coefficient before and after taxes and transfers. It finds that countries generally categorised as “liberal” in most existing typologies did the most poorly in their first-wave COVID-19 response.

## Introduction

The COVID-19 pandemic represents an extraordinary challenge in which governments have had to act in unprecedented ways. Examining the initial policy response of different countries to the appearance and spread of COVID-19 is important in its own terms, but also gives us a means of exploring the capability of governments in different countries to cope in the face of crises. Comparative analysis is a valuable tool to this end as it allows us to identify which countries have been most successful in their response, and what we can learn from them. At the end of the first wave of COVID-19, by mid-July 2020 there were sufficient data to be able to assess which countries had done best in their initial response, and what they had in common.

This paper utilises fuzzy-set qualitative comparative analysis (QCA) to explore a range of risk factors in different countries, as well as the policy response in terms of COVID-19 testing. This method is appropriate because of its focus on complex conjunctural causality in small and medium numbers of cases, but as well as this adds to the paper’s originality in using a method which is far more common in political science than in social policy. It finds that COVID-19 tests per case, rather than a simple measure of COVID-19 testing, was perhaps the most significant factor which was linked to achieving low levels of both COVID-19 cases and COVID-19 mortality. The paper then moves on, in the light of claims that particular types of government have done better than others in confronting the virus, to explore existing typologies of health and welfare systems, to see what we can learn from existing typologies of health and welfare, deriving two dimensions based on total social expenditure and the difference between market income GINI and income GINI after transfers. Locating countries across these two dimensions complements the QCA solutions, giving additional analytical depth and helping to better explain cases which appear “deviant” in terms of their causal factors or location in the typology.

## Background

Considering how different countries responded to the COVID-19 pandemic gives us key insights into their governments’ capacity to react to new global challenges. In the context of the pandemic itself, cross-national studies have the potential to help understand the inter-relationships between country-based COVID-19 risk factors, and the testing regimes they have introduced, allowing us to see whether there are patterns in the data that relate to the occurrence of COVID-19 cases and/or COVID-19 mortality. Performing this analysis also gives us the means of categorising countries in terms of these factors and their relative success, as well as exploring how these categories might relate to existing welfare and health typologies.

Because of the novelty of the pandemic, and the relatively standard international datasets that are available, not all the relevant factors relating to governmental response are easily measurable, with countries making different choices around the timing and severity of lockdown restrictions, the use and scale of test and trace regimes, whether quarantine has been imposed on the most vulnerable and on international visitors, on widespread hygiene measures including the use of masks and protective equipment have put in place, and a range of other possible factors. Projects such as the Oxford “Coronavirus Government Response Tracker”^1^ attempt to measure these factors (and others), but are still in their early stages of development. However, there are COVID-19 risk factors which are covered in established datasets exist and which can form the basis of an analysis.

In considering how well (or badly) different countries have met the challenge that COVID-19 has brought, it is useful to differentiate contextual factors which appear to contribute to COVID-19 mortality and COVID-19 cases from those which are about their government’s response. Obesity appears to be a risk factor in raising COVID-19 mortality rates, but it less clear whether it is also a factor in contracting the virus (Hastie et al., 2020). People in minority groups appear to have an increased risk of COVID-19 mortality, but it is unclear whether this is increased risk based on other contextual factors such as poor housing or social deprivation, which may also be causes of COVID-19 spread as well (Kirby, 2020). There is also good evidence for treating this topic intersectionally, especially in relation to factors such as inequality (Ragin & Fiss, 2016).We also know that people with a range of existing health conditions are especially vulnerable to COVID-19, hence the widespread use of isolation strategies for this group since the outbreak of the virus, and beyond this, there is clear evidence that the elderly in care homes have been especially affected by the virus (Chor, 2020). After 6 months, at the end of the “first wave” for most countries, it was possible to identify which countries were best at minimising infections and mortality in the first wave of the pandemic, and what they had in common. Although there were differences in the method of measuring COVID-19 cases and mortality between countries, there was sufficient in common between those measures to develop a sense of which countries had responded to this new challenge most effectively.

An Economist Intelligence Unit report published on 17 June 2020 (Economist Intelligence Unit, 2020), attempted to pull together measurable risk factors, including obesity prevalence, alongside share of population aged 65+ (both in line with existing research above), but also added an index of international arrivals to explore the relative openness of each country to infection from abroad, and from home citizens returning from overseas. This additional factor is an important addition to COVID-19 research as it gives us a sense of the initial travel-based risk different countries faced at the beginning of the crisis.

Beyond the factors directly related to COVID-19, the widespread societal response necessary to mitigate the virus’ worse effects has led to calls for wider assessments of the kinds and types of government we have in place, and to calls for a “return” to social democracy to counter the wider societal problems the virus has exposed (Johnson, 2020). This raises the question of whether the countries which have managed COVID-19 better than others fit within any welfare or health groups or clusters that exist in the present literature. The classic typology for welfare systems is provided by Esping-Andersen (1990), but has created a vast literature (Powell & Barrientos, 2011), who produce an excellent summary, as does Bambra (2007). In addition, comparative studies of health systems alone come especially from the work of Wendt (eg. Reibling, Ariaans, & Wendt, 2019; Wendt, 2009).

Against this background, the paper seeks to ask three inter-related research questions:

What patterns of national risks and measurable policy responses seem to lead to low cases and low mortality? Are there key factors which appear to be causally linked to both cases and mortality? Did particular types of welfare or health systems perform better in dealing with the challenge of COVID-19 during its first wave than others?

## Method

The first stage of the research was to gather a dataset based on key factors which were highlighted in existing COVID-19 research. OECD measures of obesity rates, and elderly population proportions (defined in terms of 65+ in the OECD measure) were used. To measure the degree of openness of countries to international visitors, in line with the Economist Intelligence Unit study, international arrivals per population data from the World Bank was then collected. The post-transfers GINI income coefficient from the OECD was then added to capture the extent to which inequality varies between countries. This measure is generally regarded as a robust measure in comparative studies of the social determinants of health (Marmot, 2015). Given the intersectional importance of inequality in relation to mortality and the risk factors for ethnic minority groups, exploring the importance of this factor in the data were clearly crucial.

The proportions of obese and elderly people, along with the index of international travel and the GINI factor represent key contextual factors which governments have to confront in dealing with COVID-19. However, it is also important to measure the policy response to the virus. To assess this total COVID-19 testing (per thousand people) was incorporated into the dataset, especially given the World Health Organisation (WHO)’s emphasis on this factor in first-wave COVID-19 (WHO, 2020). Counts of tests performed was found to be inadequate as a factor alone because some countries have been relatively unaffected by the pandemic and have, as a consequence, done relatively little testing but still been successful in terms of their outcome measures. To resolve this, a ratio between total COVID-19 tests and total COVID-19 cases was constructed, with the reasoning being that the need for testing was determined at least in part by the extent of infection in a population. This factor overlooks the precise timing of the tests, which is clearly important, but gives an insight into the infrastructural capacity of country to respond to COVID-19 by July 2020. The assumption was the higher the ratio of tests to cases, the more robust the country’s testing infrastructure, and seems to be borne out as important in research from Italy and China (Romagnani et al., 2020).

In assessing how well (or poorly) countries have responded to COVID-19, excess mortality figures probably represent the gold standard measure, but the availability of comparable excess mortality figures is limited. Instead, the best available COVID-19 deaths per million were taken,^2^ accepting that this measure is subject to some cross-national variation in measurement. However, even with some error, the mortality figures allowed us to identify countries which have done comparatively well or poorly. The date for all COVID-19 measures was 15 July 2020 – which was nearly 6 months after the WHO formally declared the virus to represent a pandemic on 30 January, and so a good time to assess the “first wave” response to the pandemic by the countries included in the sample here. At that point, there had been a stabilisation of case and mortality rates for most countries in the sample.

In addition to COVID-19 mortality, a second outcome measure based on COVID-19 cases (rather than deaths) per million population was taken. As with COVID-19 mortality, these numbers were again subject to some variations in testing regimes in different countries, as well as to variations in reporting (France have to be omitted, eg. due to not updating their figures). Using COVID-19 cases as an outcome measure, and as part of the measure of tests per case introduced a danger of duplication, but had an *r* of 0.04 only for the uncalibrated data, and 0.10 for the calibrated data, suggesting there was little relationship between the two measures.

The standard way of exploring country-level numerical data systematically would be through the use of macro-comparative quantitative methods (Babones, 2013). However, the dataset constructed here led to the use of a different technique, that of QCA, which is less used in social policy analysis, but with some notable exceptions (Haynes, Banks, & Hill, 2013; Kuhner, 2015). There are a number of reasons for the choice of QCA. First, standard statistical data depend on the variables included being substantially independent of one another. However, this is almost impossible to achieve in macro-comparative work because of regional relationships and shared histories between countries. In addition, there are likely to be a range of different routes to a good COVID-19 response rather than a single best combination of factors (Capano, Pritoni, & Vicentini, 2019), and this equifinality is difficult to assess using standard statistical methods based on linear models which assume what Ragin (2008) calls “net effects.” The paper’s solutions (presented below) include complex conjunctural causation expected which would be almost impossible to account for in terms of statistical interactional effects.

As data could be credibly represented across a range of calibrated scales, the fuzzy-set version of QCA was chosen and examined using enhanced standard analysis (Schneider & Wagemann, 2012). First, necessary conditions (those which are nearly always present in the outcome) were first looked for across the range of causal factors and outcome under investigation. Truth tables were then constructed with an initial consistency threshold of 0.8, but exploring different levels to explore the difference that this made to solution terms. Contradictory simplifying assumptions (including the inverse of necessary conditions, where they were found) were excluded from counterfactual truth table rows and so from sufficient solution terms. Finally, sufficient solutions (those where causal factors nearly always are linked to outcomes) were calculated. The solutions presented below are, again in line with enhanced standard analysis, the “intermediate” versions which include directional (theoretical) assumptions about counterfactual truth table rows, all of which are explained in the relevant solution section. Where they add to understanding, the conservative and parsimonious solutions are also mentioned in comparison. All analyses were conducted in the QCA package in R (Dusa, 2018) with the code and data available in the Supplementary Appendix.

A sample of 25 countries could be constructed from the available data with those countries also have strong representation in different welfare typologies, allowing an exploration of how well the results fit within the most significant categorisation of such systems. The “raw” data for the paper are presented in Table 1 with the causal factors (from left to right) being OBESITY (OECD obesity rate), INTARPOP (international arrivals per population), ELD (OECD proportion of people 65+), GINI (OECD income GINI coefficient), COVID-19TEST (total COVID-19 tests per thousand people), COVID-19M (COVID-19 mortality rate per 1M) and COVID-19CASE (total COVID-19 cases per 1M people).

**Table 1.** Raw data.

CASE	COUNTRY	OBESITY	INTARPOP	ELD	GINI	COVID-19TEST	COVID-19M	COVID-19CASE
1	AUS	30.40	0.36	0.16	0.33	118.49	4.24	402.00
2	AUT	21.90	3.47	0.19	0.28	77.79	78.72	2116.27
3	BEL	24.50	0.79	0.19	0.26	90.82	844.46	5417.00
4	CAN	31.30	0.56	0.17	0.31	85.13	233.11	2874.11
5	CZE	28.50	0.99	0.20	0.25	55.90	33.15	1245.78
6	DEN	21.30	2.19	0.19	0.26	211.01	105.31	2254.93
7	FIN	24.90	0.58	0.22	0.27	50.33	59.38	1317.70
8	GER	25.70	0.47	0.21	0.29	76.10	108.27	2383.82
9	GRE	27.40	2.81	0.22	0.32	36.69	18.52	372.54
10	ICE	23.10	6.49	0.14	0.26	198.66	29.30	5582.42
11	IRE	26.90	2.21	0.14	0.30	104.69	353.60	5198.68
12	ISR	26.70	0.46	0.12	0.35	127.87	42.86	4893.98
13	ITA	22.90	1.02	0.23	0.33	98.62	578.61	4024.75
14	JPN	4.40	0.25	0.28	0.34	4.44	7.78	177.96
15	KOR	4.90	0.30	0.14	0.36	27.04	5.64	264.31
16	LUX	24.20	1.64	0.14	0.33	449.59	177.32	7917.24
17	NLD	23.10	1.08	0.19	0.29	39.80	357.63	2981.70
18	NZ	32.00	0.75	0.15	0.33	89.10	4.56	248.22
19	NOR	25.00	1.06	0.17	0.26	66.42	46.67	1657.18
20	PRT	23.20	1.58	0.22	0.32	127.45	163.58	4614.33
21	ESP	27.10	1.76	0.19	0.33	82.34	607.62	5488.61
22	SWE	22.10	0.72	0.20	0.28	59.41	549.05	7525.40
23	SWI	21.20	1.21	0.18	0.30	79.12	194.93	3805.02
24	UK	29.50	0.54	0.18	0.36	105.89	662.40	4292.09
25	USA	37.30	0.24	0.16	0.39	121.70	412.28	10367.21

Before data can be analysed using fuzzy-set QCA, they must first be calibrated onto a scale between 0 (no set membership) and1 (full set membership) with 0.5 representing a point of indeterminacy. Ideally, this is achieved in relation to an external benchmark, and this was achieved in relation to factors extensively used in existing research, such as the GINI coefficient through comparison with existing work making use of that factor (especially Wilkinson & Pickett, 2010; Marmot, 2015). Beyond this, data series were calibrated by first exploring them in relation to existing research (where it existed), and comparing possible values to graphical representations, as well as to those found through cluster analysis. This allowed an in-depth understanding of the properties of the data in the sample, and the informed selection of the calibration “anchor” points for set membership. The data were then calibrated using the direct method suggested by Ragin (2008, chapter 5), and so fitted on a logistic scale. Calibration means that that some cases were close to the crossover threshold between low and high, and in one case (Germany) this had implications for the results that will be explored in greater depth in the conclusion. All calibration decisions are shown and documented in the R code available in the Supplementary Appendix. The results of the analysis follow in the next section.

## Results

This section explores the necessary and sufficient relations between five causal factors (obesity, elderly proportion, international arrivals, GINI and tests per case) first in relation to COVID-19 mortality, then COVID-19 cases outcomes, and finally an outcome made up of the logical relation COVID-19 mortality OR COVID-19 cases.

### Low COVID-19 mortality

A necessary condition is found where, given an outcome (here low COVID-19 mortality), causal conditions or combinations of them are always or nearly always occur in relation to it. In the calculation of necessary conditions for low COVID-19 mortality, the combination of high tests per case or low income inequality (TESTCASE + ~GINI) had a consistency of 0.86 and a relevance of 0.72, and high tests per case or low obesity (TESTCASE + ~OBESITY) a consistency of 0.8 and a relevance of 0.77.

The next stage of analysis is to derive a truth table. This entails making a decision about the consistency level required of the rows, or combinations of causal conditions, which are needed for that row to be included in the analysis of sufficient conditions. As a benchmark, and following the recommendations made by Ragin, an initial consistency threshold of 0.8 was taken. At that level, the following truth table is generated, but would be identical up to a consistency threshold of 0.848 or down to 0.775. Truth tables show the combinations of causal factors and outcome measure – here only the cases and truth table rows with empirical data are reproduced for space reasons. In addition to these rows, “counterfactual” rows are also calculated for combinations of outcomes which did not occur empirically with consistency measures based on the combinations of factors which did empirically occur. How these counterfactual rows are treated in terms of the calculation of solutions is explained further below (Table 2).

**Table 2.** Truth table for low COVID-19 mortality.

OBESITY	ELD	TESTCASE	INTARPOP	GINI	OUT	Consistency	PRI	Cases
0	0	1	0	1	1	0.901	0.753	KOR
0	0	1	1	0	1	0.927	0.811	ICE
0	0	1	1	1	0	0.872	0.345	LUX
0	1	0	0	0	0	0.690	0.418	BEL, SWE
0	1	0	0	1	0	0.726	0.393	JPN
0	1	0	1	0	0	0.742	0.477	NLD, SWI
0	1	0	1	1	0	0.687	0.177	ITA, PRT
0	1	1	1	0	1	0.904	0.799	AUT, DEN
1	0	0	0	1	0	0.694	0.278	ISR, USA
1	0	0	1	0	0	0.776	0.478	IRE
1	0	1	0	1	1	0.898	0.778	AUS, NZ
1	1	0	0	0	0	0.744	0.499	GER
1	1	0	0	1	0	0.597	0.069	CAN, UK
1	1	0	1	1	0	0.651	0.151	ESP
1	1	1	0	0	1	0.901	0.786	CZE, FIN
1	1	1	1	0	1	0.900	0.780	NOR
1	1	1	1	1	1	0.849	0.591	GRE

Abbreviation: PRI, proportional reduction in consistency.

There is a row in the truth table above that has a consistency above 0.8, but which is excluded from being taken forward to the solution (so OUT = 0), ie. the third truth table row, which has a consistency of 0.872 (LUX). Although that row has a consistency above 0.8, it has a proportional reduction in consistency (PRI) below 0.5, and so potentially appears in both the low mortality and high mortality solution – and so the decision to exclude it from the low mortality solution was taken.

Having carefully examined the truth table, the next stage of analysis is reached, where sufficient solutions are calculated. This is probably the most important stage of QCA as sufficient solutions are effectively the pathways to the achievement of the outcome we are interested in – here low COVID-19 mortality. Sufficient solutions are those where, starting from the combinations of causal factors, the outcome is always, or nearly always found. The following sufficient solution was generated with directional expectations, in line with existing research, of low elderly proportion, low GINI coefficient and low international arrivals (Table 3).

**Table 3.** 

Solution term	Consistency	PRI	Coverage	Unique coverage	Cases
TESTCASE*~INTARPOP	0.913	0.853	0.510	0.141	AUS, CZE, FIN, KOR, NZ
TESTCASE*~GINI	0.936	0.883	0.478	0.007	AUT, CZE, DEN, FIN, NOR, ICE
OBESITY*ELD*TESTCASE	0.885	0.786	0.376	0.024	CZE, FIN, GRE, NOR

Solution consistency 0.93, coverage 0.65.

Abbreviation: PRI, proportional reduction in consistency.

The low COVID-19 mortality sufficient solution has three pathways.

The first pathway has the largest unique coverage and covers five cases, combining high testing per case and low international arrivals. The second pathway covers six cases and combines high testing per case with low GINI. The third pathway combines high testing per case with both high obesity and high elderly population, and covers four cases. All three pathways incorporate the TESTCASE necessary condition.

In these solutions, there are no cases deviant for consistency, and the overall solution has a high consistency (0.93). However, there are three countries which achieved low COVID-19 mortality in the first wave, but did appear in these solution pathways – GER, ISR and JPN – and are therefore cases which are “deviant” for coverage. The discussion section below will pick up on all deviant cases and explore them in greater detail to account for possible reasons for their non-fit with solution terms.

In QCA, three sufficient solutions are calculated, depending on the assumptions made about the “counterfactual” data combinations. In the solution above (the intermediate one), only counterfactual rows which met the specified directional (theoretical) expectations were included. In addition to the intermediate solution, a conservative solution is calculated based on empirical data only, and a parsimonious solution calculated based on including all counterfactual rows.

The conservative solution was more complex than the intermediate one, with the first solution pathway adding GINI*~ELD but having the same countries included, the second pathway splitting into two more complex pathways – one AUT, DEN and ICE, and the other for CZE, FIN and NOR (both of which are subsets of the pathway above), and the third pathway having a more complex subset with AUS, KOR and NZ included, but GRE and NOR being split into another pathway solution. The parsimonious solution, based on all possible counterfactuals, offered four alternative solutions, but with one identical to the intermediate solution above. As such, the intermediate solution presented above seems to be robust across the different assumptions about counterfactual truth table rows.

### COVID-19 mortality OR COVID-19 cases

As well as calculating solutions for low COVID-19 mortality, solutions for low numbers of COVID-19 cases were also calculated. These solutions proved to be more complex than those for mortality, and while important, are included in the Supplementary Appendix only as they are perhaps less immediately important than those for mortality. Calculating sufficient relations for COVID-19 cases also allowed solutions to be generated for countries that had experienced both low numbers of cases and low mortality. It is to those solutions – representing the countries that responded most successfully to COVID in its first wave – that the paper now turns.

Fuzzy logic, to achieve the set of countries with outcomes with low scores for both mortality and cases, requires a calculation of COVID-19 mortality OR CASES, which means the outcome takes the highest value from either set. This has the effect of requiring cases in the high set to have either high COVID-19 mortality or high case numbers (or both), whereas those in the low set must have both low COVID-19 mortality and low case numbers. As such, using the fuzzy OR method is most demanding method of joining the two outcomes, requiring countries in the low set in both mortality and case outcomes.

For this outcome, a combination of high tests per case or low GINI (TESTCASE+~GINI - consistency 0.90, relevance 0.64) and high tests per case or low obesity (TESTCASE+~OBESITY – consistency 0.88, relevance 0.70) were important in terms of both their measures and fit to existing research, and so were included as necessary conditions. The truth table (included in the Supplementary Appendix) was produced with a consistency threshold of 0.788, so slightly below the ideal benchmark 0.8, but lowered to include AUS, NZ and KOR in the solution term, all of which had a PRI score above 0.5, and were clearly in the low outcome set. Reducing the consistency threshold slightly increased the coverage of the sufficient solution, while not introducing any deviant cases for consistency, and was justified in terms of the process of moving back and forth between cases and data (Ragin, 2014). The sufficient solution was produced with directional expectations of low obesity, low elderly proportion, low GINI and low international travel, in line with previous solutions and existing research, but again only produced small variations by varying these expectations (Table 4). It was as follows:

**Table 4.** 

Solution	Consistency	PRI	Coverage	Unique coverage	Cases
TESTCASE*ELD	0.875	0.766	0.522	0.131	AUT, CZE, DEN, FIN, GER, NOR
TESTCASE*~INTARPOP	0.848	0.744	0.586	0.195	AUS, CZE, FIN, KOR, NZ

Solution consistency 0.872, Coverage 0.718.

Abbreviation: PRI, proportional reduction in consistency.

The first solution pathway combines high testing per case and a high elderly population, and the second pathway high testing per case and low international travel. Both pathways have high coverage, and around the same unique coverage, along as incorporating the same necessary condition (TESTCASE).

There are no cases deviant for consistency in this solution, but two cases deviant for coverage (GER and JPN) – so represented cases which had low COVID-190 mortality or cases, but were not included in the solution. The parsimonious solution was identical to the intermediate one with the conservative solution having four pathways, all of which are subsets of the two solution terms above.

**Figure 1.**
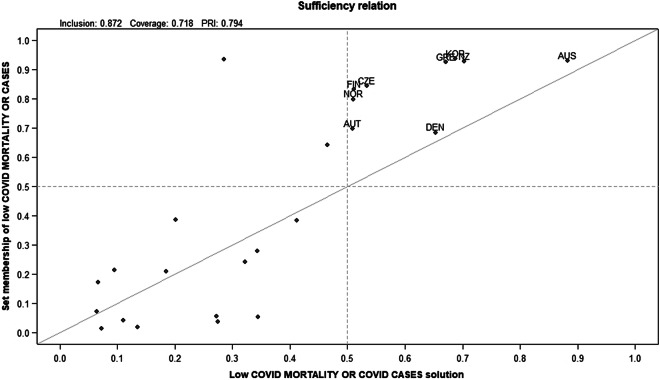
Sufficient solution for low COVID-19 mortality OR low COVID-19 cases.

Given the importance of this solution term – representing the very best performing countries in terms of minimising both COVID-19 cases and COVID-19 mortality, the solution can is also represented graphically in Figure 1.

Figure 1 shows the relation between the sufficient solution for low COVID-19 mortality or COVID-19 case to mid-July, and the set membership of countries with low COVID-19 mortality of cases. The countries with high low COVID-19 mortality or low cases are labelled. The countries which are in the solution set are in the top right of the chart, indicating that there are no cases deviant for consistency. The chart also allows us to see the most typical cases – the ones which have the closest match of solution and data – as they are closest to the 45-degree line – DEN and AUS, with DEN most closely matching the first solution pathway, and AUS the second. These two countries, then, represent the nearest to ideal types in terms of their “match” between solutions, mortality and cases.

## Discussion

To summarise the solutions presented above, the sufficient solution for low COVID-19 mortality as an outcome has three pathways, all of which high testing per COVID-19 case in common, then combined with low international travel, low income inequality or high obesity and high elderly proportion. The factor the solutions have in common, however, is high tests per COVID-19 case, emphasising the importance of testing (in relation to case numbers) and confirming its key role as a necessary condition. The low COVID-19 mortality OR COVID-19 sufficient solution has two pathways only. The first combines high tests per case with a high elderly population (in common with the first pathway of the low cases solution), and the second high tests per case combined with low international travel (in common with the first solution pathway for low COVID-19 mortality). From these solutions, it is hard to get away from the importance of testing in proportion to cases, but in the context that this factor forms sufficient solutions alongside different causal factors to generate the pathways to strong COVID responses, emphasising the importance of treating causation conjuncturally as QCA does.

It is also important to consider cases which were “deviant” in the sufficient solutions. These are cases which either appeared in solution terms when they did not meet the outcome (deviant for consistency) or which did not appear in solution terms when they did achieve the outcome (deviant for coverage). Data will always be imperfect, and no simple model can completely capture reality – especially in the flux of a global crisis. Exploring deviant cases gives additional insight into both our cases and the solution patterns that have been identified. There were no cases deviant for consistency in the results presented above, but some that were deviant for coverage. In the low COVID-19 mortality solution GER, ISR and JPN were in this category – and so have achieved low COVID-19 mortality, but not through the same route as the QCA solution pathways. GER and JPN were also deviant for coverage for low COVID-19 mortality OR cases. It is therefore worth considering these cases in additional depth.

Despite being internationally lauded in terms of its testing regime, Germany falls just within the just within the calibrated measure of having low tests per case, and so is a marginal case of deviancy. At the same time, however, in may also have taken a more sophisticated approach to testing than in other countries – whereas the measure of tests per case gives a wide “net” to catch cases, a sophisticated test and trace system might allow fewer tests to be carried out, but ensure they are being focussed on the right people (Beaumont & Connolly, 2020; Desson et al., 2020).

Japan is unusual in that it did not impose a lockdown until April, and so a month after many other countries included here, but may have had advantages in terms of social factors such as more routine wearing of masks and more socially distant customs such as bowing (rather than hand-shaking or kissing). Although Japan imposed lock-down later than other nations, it was quicker than many western nations in banning mass gatherings, and this may have also prevented large-scale transmission events. Japan looks very much more of an obviously outlier case in terms of its response to COVID-19 (Fukushima, 2020).

Finally, it is clear from the international COVID-19 tracker^3^ as well as international media coverage, that Israel represents a very unusual case, with a low COVID-19 mortality during its first wave, but a relatively high number of COVID-19 cases. However, in terms of the 6-month timescale the paper covers, it appeared to be entering a second peak of cases earlier than other cases in the sample (Schulman, 2020), perhaps due to reopening its economy too early.

As such, of the three cases deviant for coverage in the solutions above, Germany appears to be missing from sufficient solution terms because of its more focussed approach to testing, with Japan appearing more of an outlier where further research would be extremely useful, and Israel out of synchronisation with other countries in its

### COVID-19 solutions and health and welfare typologies

To take the analysis further, the QCA results can be compared to those from existing welfare and health typologies to explore the relationship between the two, and assess whether particular types of welfare and health systems appear to be associated with stronger first-wave responses to COVID-19. It would be virtually impossible to explore every welfare and health typology given the explosion of work since the publication of Esping-Andersen (1990). However, Bambra (2007) produces an important summary, to which further work, including that of Wendt in his categorisations of health systems (Reibling, Ariaans, & Wendt, 2019; Wendt, 2009), can be added. The locations of countries in those typologies can then be compared to their inclusion in the low set for COVID-19 mortality and COVID-19 cases (a full table of this comparison is included in the Supplementary Appendix).

The typologies which best fit the countries in the low COVID-19 mortality OR COVID-19 cases were those from Castles and Mitchell (1993), Bonoli (1997) and Pitruzzello (1999). These typologies are all based on general characteristics of welfare systems, suggesting that impact of the pandemic goes wider than the health system alone. Within these three typologies, the first two especially put an emphasis on welfare expenditure as one category, alongside a measure of the extent of societal redistribution. Working through the detail of these typologies, and updating them for the most recent data available allowed two welfare dimensions to be mapped against one another based on their underlying logics, and based on updated data from the most recent year available.

**Figure 2.**
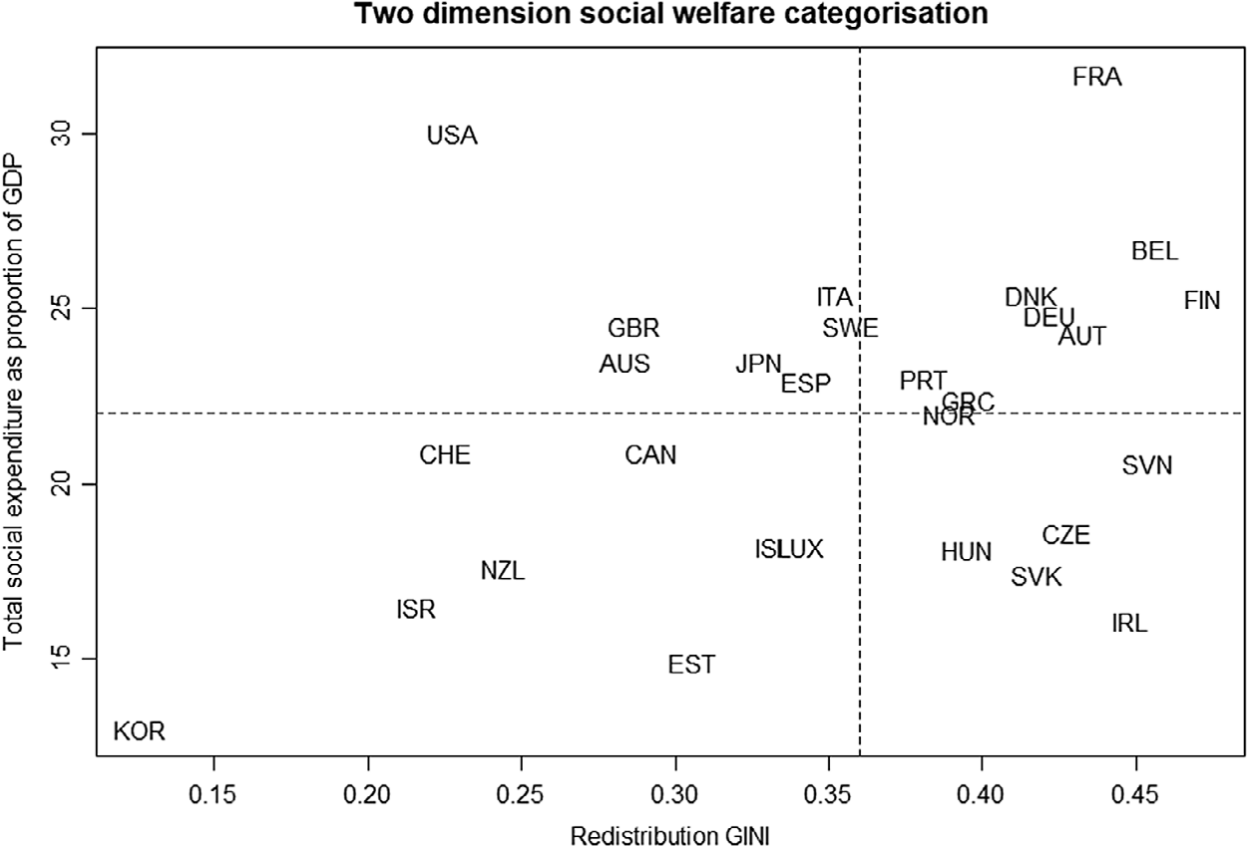
A two-dimensional social welfare categorization.

The first dimension considered was social expenditure (both public and private) as a proportion of GDP to capture the OECD’s insight (, 2014) that to capture the full extent of welfare spending we need to consider both public and private sources. Using total social expenditure comes with the potential problems of the fairness and affordability of provision, however, so the second dimension measures, following Castles and Mitchell (1993), the proportional difference between the GINI income coefficient for market income, and the GINI coefficient after taxes and transfers, to assess the redistributive extent of different welfare systems. This dimension also links to the consistent appearance of the GINI factor in the QCA solution terms above. All countries for which consistent measures could be found were included, leading to the inclusion of some not in the COVID-19 solutions above. Finally, the median values for each dimension were taken, and which divided the countries into four sectors which can be diagrammed as follows:

Figure 2 and its underlying data, in turn, can be related to their position in the solution set (above) of COVID-19 mortality OR COVID-19 cases. This yielded Table 5, with countries in the high set having “+” next to their names and those in the low set having “~.”

**Table 5.** Two-factor typology and “fit” with the calibrated scores for high or low COVID-19 mortality OR COVID-19 cases.

Redistribution (GINI)/total social expenditure	Low total social expenditure	High total social expenditure
Low redistribution	KOR ~	Low G/high T
	ISR +	AUS ~
	NZL ~	UK +
	EST N/A	JPN ~
	ISL +	ESP +
	LUX +	ITA +
	CAN +	SWE +
	CHE +	USA +
High redistribution	IRL +	PRT +
	SVK N/A	GRC ~
	HUN N/A	NOR ~
	CZE ~	AUT ~
	SVN N/A	GER ~
		DNK ~
		BEL +
		FIN ~
		FRA N/A

From Table 5, countries with both high redistribution and high total social expenditure have the strongest first-wave COVID-19 outcomes (with NOR being a borderline case just falling within this group). This category of countries includes some countries which are generally categorized as Social Democratic (AUT, BEL, DNK, NOR) but also some others categorized as Conservative (FIN, FRA, GER), as well as GRC and PRT from “Southern” group (see eg. (Bambra, 2007). There are, however, no countries usually categorised as “Liberal.” There are also two countries that appear to have a favourable welfare context for handling COVID-19, but which have done worse than might have been anticipated – BEL and PRT, with Belgium especially interesting because of its very high-profile problems in dealing with COVID-19.

Examining the governmental response tracker for COVID-19 as well as contemporary news coverage, in Belgium the response to the virus appears to have become highly politicised (Galindo, 2020), with the government claiming its higher death rate is due to more rigorous reporting of deaths than in other countries (de Block, 2020). However, Belgium also appears as having amongst the highest death rate in the EIU report (which uses excess mortality rather than COVID-19-specific mortality, as in this paper), and there appear to have been clear failings in terms of the provision of PPE that mirrored those of the UK, another country with a high mortality rate. Equally, neither Belgium nor Portugal can claim to have a test per case ratio that which would put it in the high set for this factor, and the importance of this causal factor in the solutions above has already been made clear. It would seem, from this analysis, that Belgium and Portugal fell short in their first-wave COVID-19 response, especially in relation to testing, and despite the relative advantages that their social welfare systems held for them.

Looking at the combination of low redistribution but high total social expenditure, countries in this category tend to produce low outcomes, except for AUS and JPN, with the former notoriously difficult to categorise because of its combination of its liberal economy with a more extensive welfare system than we might expect, and the latter (as noted above) a case deviant for both consistency and coverage in our analysis above, underlying its unusually successful (in terms of COVID-19) pattern of causal factors.

The combination of low redistribution and low total social expenditure includes a majority of countries with relatively poor COVID-19 outcomes, except for NZ and KOR, which appear along with AUS, CZE and FIN, in the low COVID-19 mortality OR COVID-19 case solution and have the combination of high tests per case and low international arrivals. NZ, KOR, AUS, CZE and FIN therefore have these risk factors in their favour, both of which repeatedly appeared in the QCA solution terms of strongly-performing countries, despite the less helpful contexts their welfare systems appear to create.

Finally, in the combination of high redistribution but low total social expenditure, there are only two countries included in the COVID-19 case and mortality data, so this combination offers an opportunity for further research. However, it is worth noting that three of the five countries here are outliers in terms of most OECD health and welfare measures, and so whether they can form part of a wider systematic comparison in terms of the factors identified here is perhaps more open to question. They do form a clear fourth cluster or category in terms of their position in relation to the redistribution and total social expenditure typology here though.

## Conclusion

Considering how well countries responded to challenge of a global pandemic gives us insights not only into their ability to put in place pandemic-specific measures (such as testing) but also how key contextual factors have worked in relation to them. Placing the QCA results in the context of a two dimensional mapping of countries derived from existing welfare typologies, added additional depth as well as linking the solutions to existing typology research.

The TESTCASE factor, COVID-19 tests per case, has a strong claim to be the most important causal factor for first-wave response to COVID-19, being both conceptually important as it shows the importance of the testing regime needing to be in proportion to the number of cases, as well as empirically central to the solutions that were generated. TESTCASE appears in all the solution pathways for low COVID-19 mortality, in two of the pathways for low COVID-19 cases, and in both solution pathways for low COVID-19 mortality OR COVID-19 cases. As such, it is not a high volume of testing per se that was linked to lower levels of COVID-19 cases or mortality, but high numbers of test *per case* – and that is a key distinction. High numbers of tests per case suggest a governmental response designed to test not only those with the virus, but also to prevent further spread. The test per case factor may also be a proxy for the ability of governments to quickly respond to a novel challenge. Putting in place a robust and largescale testing regime is not a trivial challenge and is one that countries responded to with different degrees of success. It will be fascinating to see if the countries that were able to meet this challenge are also those best able to respond to future global societal challenges.

The addition of international arrivals appears in solution terms throughout the paper, and so appears a useful addition, even though it has not centrally appeared in existing COVID-19 research, as it. The international arrivals factor appears in the first solution pathway for low COVID-19 mortality (as low), as both low and high pathways in relation to COVID-19 cases (and in all but one solution pathway), and in the solution pathway for low COVID-19 mortality OR COVID-19 cases (as low).

It is clear that achieving a strong first-wave COVID-19 response does not mean countries have necessarily achieved subsequent success in dealing with the virus as well. However, the results do give us an indication of which countries did manage to most successfully meet the initial challenge the pandemic offered, and this presents an opportunity for future research exploring its relationship to both subsequent response to COVID-19, as well as to other novel crises which, if the “risk society” thesis is correct (Beck, 1992), are set to become a feature ever-more present in our lives.

After deriving its QCA results in relation to COVID-19, the paper compared them to existing typologies of welfare and health, from which dimensions based on total social expenditure and differences in market and post-transfer GINI coefficients were derived. A mapping of countries in the sample based on those dimensions was constructed, with a clear relationship between them and the COVID-19 outcome measures. Exploring the positioning of countries across these two dimensions and in relation to their QCA solutions makes clear those which are unusual in their positioning (as for AUS, NZ and KOR), or by their appearance in QCA solutions as deviant cases (such as JPN) for coverage, or which we might have expected to have handled COVID-19 better than they did (such as BEL). For countries which have done worse than we might expect from their position in the typology (BEL and PRT), low testing per case may well be the decisive factor, despite those countries having a social welfare system shared with other systems with much stronger COVID-19 responses. Overall, it cannot be concluded that the countries most likely to have effectively responded to COVID-19 in its first-wave were especially social democratic, conservative, or even southern, but only by exception were they liberal.

## Footnotes

1 https://www.bsg.ox.ac.uk/research/research-projects/coronavirus-government-response-tracker.

2 https://ourworldindata.org/coronavirus is an excellent source with figures from there being cross-checked against those reported by the World Health Organization.

3 https://www.bsg.ox.ac.uk/research/research-projects/coronavirus-government-response-tracker.

## Supplementary material

For supplementary material accompanying this paper visit http://dx.doi.org/10.1017/ics.2021.6. click here to view supplementary material
