# Supplementary material for: Comparing country risk and response to COVID-19 in the first 6 months across 25 Organisation for Economic Co-operation and Development countries using qualitative comparative analysis
Source: Journal of International and Comparative Social Policy. 2021 Mar 30:1–15. doi: 10.1017/ics.2021.6 (PMC8047397; doi:10.1017/ics.2021.6)
Supplement: Supplementary file 1 [file S2169976321000061sup001.docx]

**Supplemental materials for online**

**Existing welfare typologies**

| **Citation** | **Type** | **Types/Clusters** | | | | |
| --- | --- | --- | --- | --- | --- | --- |
| Esping-Andersen (1990) | 18 countries  Decommodification  Social stratification  Public-private mix | **Liberal**  Australia  Canada  Ireland  NZ  UK  USA | **Conservative**  Finland  France  Germany  Japan  Italy  Switzerland | **Social democratic**  Austria  Belgium  Netherlands  Denmark  Norway  Sweden |  |  |
| Castles and Mitchell (1993) | 14 countries  Aggregate welfare expenditure  Benefit equality | **Liberal**  Ireland  Japan  Switzerland  USA | **Conservative**  Germany  Italy  Netherlands | **Non-Right hegemony**  Belgium  Denmark  Norway  Sweden |  | **Radical**  Australia  NZ  UK |
| Bonoli (1997) | 16 countries  Social expenditure % GDP  Social expenditure financed via contributions | **British**  Ireland  UK | **Continental**  Belgium  France  Germany  Luxembourg  Netherlands | **Nordic**  Denmark  Finland  Norway  Sweden | **Southern**  Greece  Italy  Portugal  Spain  Switzerland |  |
| Pitzruzzello (1999) | 18 countries  Cluster analysis of decommodification | **Liberal**  Canada  Ireland  UK  USA | **Christian Democrat**  Belgium  Netherlands  Germany  France  Italy  Switzerland | **Social democratic**  Belgium  Denmark  Norway  Sweden | **Conservative-Bismarckian**  Austria  Finland  France  Italy  Japan | **Radical**  Australia  NZ |
| Bambra (2005) | 18 countries  Healthcare services and decommodification | **Liberal**  Australia  Japan  USA | **Conservative**  Austria  Belgium  Canada  Denmark  France  Italy | **Social Democratic**  Finland  Norway  Sweden | **Conservative subgroup**  Germany  Switzerland  Netherlands | **Liberal subgroup**  Ireland  UK  NZ |
| Bambra (2007) | Defamilisation and welfare regimes – cluster analysis | Australia  USA | Canada  Finland  UK | Norway  Sweden | Austria  Belgium  France  Germany  Netherlands  NZ  Portugal  Switzerland | Italy  Japan  Unclear  Denmark  Ireland  Greece  Spain |
| Castles and Obinger (2008) | 20 countries, cluster analysis – k=5 version | **English**  **Speaking**  Canada  UK  Australia  USA  Ireland | **Continental (North)**  Austria  Germany  Belgium  Finland  France  Netherlands  Portugal | **Scandinavian**  Sweden  Denmark  Finland  Norway | **Continental (South)**  Italy  Spain  Greece | **English ‘stepchildren’**  Japan  Switzerland |
| Castles and Obinger (2008) | 20 countries, cluster analysis – k=3 version | **English (liberal)**  Canada  UK  Australia  USA  Ireland  Japan  Switzerland | **Continental (conservative)**  Austria  Germany  Belgium  Finland  France  Netherlands  Portugal  Italy  Spain  Greece | **Scandinavian (social democratic)**  Sweden  Denmark  Finland  Norway |  |  |
| Wendt (2014) | 32 countries  Cluster analysis of health data |  | **Low spend, high public, low OOP, access control very high**  Australia  Czechia  Denmark  Estonia  Hungary  Ireland  Italy  Netherlands  Poland  Slovakia  Slovenia  UK | **Average spend, high public, low OOP, access strict**  Finland  Iceland  Portugal  Spain  Sweden | **High spend, highest public, low OOP, free choice**  Austria  Belgium  Canada  France  Germany  Japan  Luxembourg  NZ | **Unclassified**  Greece  Korea  Norway  Switzerland  USA |
| Reibling et al (2019) | 29 countries  Cluster analysis of health data including outcomes | **Supply and performance private**  Switzerland  USA | **Supply and choice public**  Australia  Austria  Belgium  Czechia  Germany  France  Ireland  Iceland  Luxembourg  Slovenia | **Performance and Primary Care public**  Finland  Japan  Korea  Norway  NZ  Portugal  Sweden | **Regulation public systems**  Canada  Denmark  Spain  Italy  Netherlands  UK | **Low supply and low performance mixed**  Estonia  Hungary  Poland  Slovakia |
| Greener (2020) | 11 countries, healthcare financing and outcomes – not mutually exclusive categories | **High spend, high OOP, low Voluntary insurance**  Norway  Switzerland  Sweden |  | **High Gov, Low Voluntary insurance**  Germany  Netherlands  NZ  Norway  Sweden  UK | **High spend, high GOV, low OOP**  France  Germany  Netherlands | **Low spend, low Gov, high OOP, high Voluntary insurance**  Australia |

Green = low set of countries

Red = high set of countries
